# Prevalence of anemia and its associated factors in human immuno deficiency virus infected adult individuals in Ethiopia. A systematic review and meta-analysis

**DOI:** 10.1186/s12878-018-0127-y

**Published:** 2018-11-12

**Authors:** Ayenew Negesse, Temesgen Getaneh, Habtamu Temesgen, Tesfahun Taddege, Dube Jara, Zeleke Abebaw

**Affiliations:** 1 Department of Human Nutrition and Food Sciences, College of Health Science, Debre Markos University, P.O. Box 269, Debre Markos, Ethiopia; 2 Department of Midwifery, College of Health Science, Debre Markos University, P.O. Box 269, Debre Markos, Ethiopia; 3 Ethiopia Field Epidemiology and Laboratory Training Program (EFELTP) Resident, University of Gondar, P.O. Box 196, Gondar, Ethiopia; 4 Department of Public Health, College of Health Science Debre Markos University, P.O. Box 269, Debre Markos, Ethiopia; 5 School of Public Health, College of Health Science, Addis Ababa University, Addis Ababa, Ethiopia; 6 Department of Health Informatics, University of Gondar, P.O. Box 196, Gondar, Ethiopia

**Keywords:** Anemia, Adult individuals, Pooled prevalence, Ethiopia, HIV/AIDS

## Abstract

**Background:**

Anemia is a common hematologic disorder among human Immunodeficiency virus (HIV) infected adult Individuals. However, there is no concrete scientific evidence established at national level in Ethiopia. Hence, this review gave special emphasis on Ethiopian HIV infected adult individuals to estimate pooled prevalence of anemia and its associated factors at national level.

**Methods:**

Studies were retrieved through search engines in PUBMED/Medline, Cochrane Library, and the web of science, Google and Google scholar following the Preferred Reporting Items for Systematic Review and Meta-Analysis Protocols (PRISMA-P). Joanna Briggs Institute Meta-Analysis of Statistical Assessment and Review Instrument (JBI-MAStARI) was used for critical appraisal of the included studies. Random effects meta-analysis was used to estimate the pooled prevalence of anemia and associated factors at 95% Confidence interval with its respective odds ratio (OR). Meta regression was also carried out to identify the factors. Moreover, Sub-group analysis, begs and egger test followed by trim-and-fill analysis were employed to assess heterogeneity and publication bias respectively.

**Result:**

A total of 532 articles were identified through searching of which 20 studies were included in the final review with a total sample size of 8079 HIV infected adult individuals. The pooled prevalence of anemia was 31.00% (95% CI: 23.94, 38.02). Cluster of Differentiation 4 (CD4) count <= 200 cells/μl with OR = 3.01 (95% CI: 1.87, 4.84), World Health Organization (WHO) clinical stage III&IV with OR = 2.5 (95% CI: 1.29, 4.84), opportunistic infections (OIs) with OR = 1.76 (95% CI: 1.07, 2.89) and body mass index (BMI) < 18.5 kg/M^2^ with OR = 1.55 ((95% CI: 1. 28, 1.88) were the associated factors.

**Conclusion:**

This review demonstrates high prevalence of anemia among HIV infected adults. Low CD4 count, WHO clinical stage III&IV, OIs and low level of BMI were found to have significant association with the occurrence of anemia. Therefore, the responsible stockholders including anti retro viral treatment (ART) clinics should strengthen the system and procedures for the early diagnosis of opportunistic infection and screening of underlying problems. There should be also early screening for OIs and under nutrition with strict and frequent monitoring of HIV infected individuals CD4 count.

## Background

Anemia is occurs when the number of red blood cells or their oxygen carrying capacity becomes insufficient to meet the physiologic condition [1]. Anemia is also common hematologic disorder among HIV infected individuals [2] with impact on quality of life and clinical outcomes among these individuals [3].

This hematologic disorder may be attributed by HIV infection itself (the incidence is increased from asymptomatic to final Acquired Immuno Deficiency Syndrome (AIDS) stage) [4],OIs, gastrointestinal bleeding, nutritional deficiencies and erythropoietin depletion [5–7]. The complex interplay of all those factors cause life threatening symptoms, which also increases the hazard of mortality among HIV infected individuals [5, 8].

A compressive global estimate of anemia in the year from 1990 to 2010 was 32.9%, resulting in 68.4 million years lived with disability (YLD) [9]. Another systematic analysis of global anemia in the year 1990–2013 also reported the high burden of anemia in which developing countries account nearly 90 % of all anemia-related disability [10].

The prevalence of anemia among HIV infected adult individuals ranges from 20 to 80% and associated with high burden of morbidity and mortality [11]. Anemia among HIV infected individuals is a well-documented phenomenon and need to be the focus area of research to determine the prevalence and its predictors in a specific country [11, 12].

Based on this recommendation, a number of researches were done and reported that the prevalence of anemia among HIV infected adult individuals was:11.2% at northwest parts of Ethiopia in 2014 [3], 11.4% at Addis Ababa in 2018 [13],11.5% at northwest parts of Ethiopia in 2014 [14], 12% at southern parts of Ethiopia in 2005–2010 [15],14.3% at Addis Ababa in 2011/2012 [16], 22.6 at southern parts of Ethiopia in 2016 [17], 23%at northwest parts of Ethiopia in 2015 [18], 23.1% at Southwest parts of Ethiopia in 2012 [19], 25% at Northwest parts of Ethiopia in 2016 [20], 32.48% at Addis Ababa in 2011/2012 [21], 33% at Addis Ababa in 2008–2012 [22], 34.4% at northeast parts of Ethiopia in 2010–2013 [23], 35% at northwest Ethiopia in 2011/2012 [24], 41.2% at Eastern parts of Ethiopia in 2014 [25], 43% at northwest parts of Ethiopia in 2012/2013 [26], 51.74% at Gambella region in Southwest parts of Ethiopia in 2014 [27], 53.3% in Southern parts of Ethiopia in 2016–2013 [3] and 70.1% at northwest parts of Ethiopia in 2011/2012 [28].

The above individual studies revealed that the findings pertaining to the prevalence of anemia are inconsistent across regions and had variations over time. Similar to the prevalence of anemia, predictor variables were also inconsistent among the aforementioned studies. In addition to this gap, lack of documented data on pooled prevalence of anemia and its associated factors among HIV infected adult individuals at national level hinders program managers to design and implement effective strategies.

With the existing meager evidence in developing countries, we set out to explore the evidence on national pooled prevalence of anemia, and to our knowledge, found no published systematic review and meta-analysis so far on this topic to help guide decision-making. Therefore, this review would summarize the available evidence on the pooled prevalence of anemia among HIV infected adult individuals and associated factors.

## Methods

### Search strategy

Initially databases were searched for same systematic review and meta-analysis to avoid duplications. First DARE database (http://www.library.UCSF.edu) was explored in an attempt to confirm whether systematic review or meta-analysis exists and for availability of ongoing projects related to the topic. We also searched the three Trials Registries: ICTRP, Clinical Trials.gov and PROSPERO (searched January 2018). By using this method, it was confirmed that there was no any review and meta-analysis was conducted in similar to with this topic.

We systematically reviewed and analyzed published research articles to determine the pooled prevalence of anemia and its associated factors among HIV infected adult individuals in Ethiopia. To identify published articles, major databases such as PUBMED/MEDLINE, WHOLIS, Cochrane library, web of science, Google and Google Scholar were used accordingly. In addition, reference lists of relevant studies were identified and the full-text articles reviewed for inclusion. The key term used for PubMed search were “Prevalence” OR “incidence” AND “Anemia” AND “HIV” OR “AIDS” AND “Ethiopia”. The Search terms were pre-defined to allow a comprehensive search strategy that included all fields within records and Medical Subject Headings (MeSH terms) was used to help expand the search in advanced PubMed search. This study also used Boolean operator (within each axis we combined keywords with the “OR” operator and we then linked the search strategies for the two axes with the “AND” operator). We followed the Preferred Reporting Items for Systematic Reviews and Meta-Analyses (PRISMA) guideline during the systematic review [29].

### Eligibility criteria

We reviewed abstracts from initial search using defined inclusion and exclusion criteria.

### Inclusion criteria

#### Study scope

All studies which determine the prevalence of anemia and which identify the associated factors of it among HIV infected adult individuals were included under this systematic review and meta-analysis.

#### Study design

Both cross sectional and cohort study designs were included.

#### Study setting

Both community and health institution level studies.

#### Language

All articles published in English language were included.

#### Population

All HIV infected adult individuals in Ethiopia.

#### Publication and publication year

Published articles until May 05/2018 were included.

### Exclusion criteria

Based on the eligibility criteria, we read their titles and abstracts. If studies are relevant for our review, we examined the full texts. Those papers which didn’t fully accessed at the time of our search process were excluded from this review after contact was attempted with the principal investigator through email at least two times. After reading the abstracts, if the studies are relevant to our review, we examined the full texts. The reason for the exclusion of these articles is that we are unable to assess the quality of each article in the absence of their full texts. Studies which didn’t report specific outcomes for anemia and associated factors quantitatively were also excluded from this systematic review and meta-analysis. Moreover, studies with poor quality as per settled criteria and review articles were also excluded from the review.

### Data abstraction

The Database search results were combined and duplicate articles were removed manually using Endnote (version X8). Data were extracted by two authors using a standardized data extraction spread sheet. The data extraction spreadsheet was piloted on 7 randomly selected papers and modified accordingly. Data extraction sheet included study characteristics such as: (1) Authors’ name, year, region, study or publication year, study design, study setting and study population; (2) incidence or prevalence of anemia (3) data extracted on sex, OIs, CD4 counts, WHO clinical stages, residence, their nutritional status, residence and their history of malaria infestation; (4) studies’ quality score, sampling technique, study area, estimating technique of anemia using Hemoglobin measurement and their mean age of the study participants were also extracted from each individual studies.

### Quality assessment (appraisal) of the individual studies

The Database search results were combined and duplicate articles were removed manually using Endnote (version X8). Joanna Briggs Institute Meta-Analysis of Statistics Assessment and Review Instrument (JBI-MAStARI) adapted for both cross sectional and follow up study design was used [30]. Three independent reviewers critically appraised each individual paper. Disagreements between those reviewers were solved by discussion. If not third reviewer was involved to resolve inconsistencies in between the two independent reviewers. Studies which score five and above were included in the final systematic review and meta-analysis.

### Outcome measurements

This review has two main outcomes. The primary outcome was prevalence of anemia among HIV infected adult individuals which was calculated as number of adult HIV infected individuals who experienced anemia divided by total adult HIV infected individuals who were at risk of developing anemia and multiplied by 100%. The second aim of this review was to identify predictors of anemia among adult HIV infected individuals in Ethiopia.

### Data analysis and synthesis

The extracted data were entered into computer using excel sheet and imported to STATA 14 for analysis. Evidence of publication bias was assessed using both egger’s test and begg’s test with *p*-value of less than 0.05 as a cut of point to declare the presence of publication bias [31, 32]. Heterogeneity across studies was checked using the inverse variance (I^2^) with Cochran Q statistic at 25, 50 and 75% as low, moderate and sever heterogeneity respectively [33]. Forest plot was also used to visualize the presence of heterogeneity. *P* value less than 0.05 was also used to declare the presence of heterogeneity across studies. Potential differences between studies were explored by sub-group analyses and Meta regression. The impact of heterogeneity across studies on the meta-analysis was quantified by I-square statistic (TAU) and a cutoff point of 50% was used to declare substantial heterogeneity. The effect size of categorical data was expressed using odds ratio.

### Measures of effect and reporting

PRISMA flow diagrams used to summarize the study selection process. Random effects (DerSimonian and laird) model was used during analysis and Odds Ratios with their 95% CI were used to present the pooled effect sizes.

## Results

### Selection of studies

**Fig. 1:**
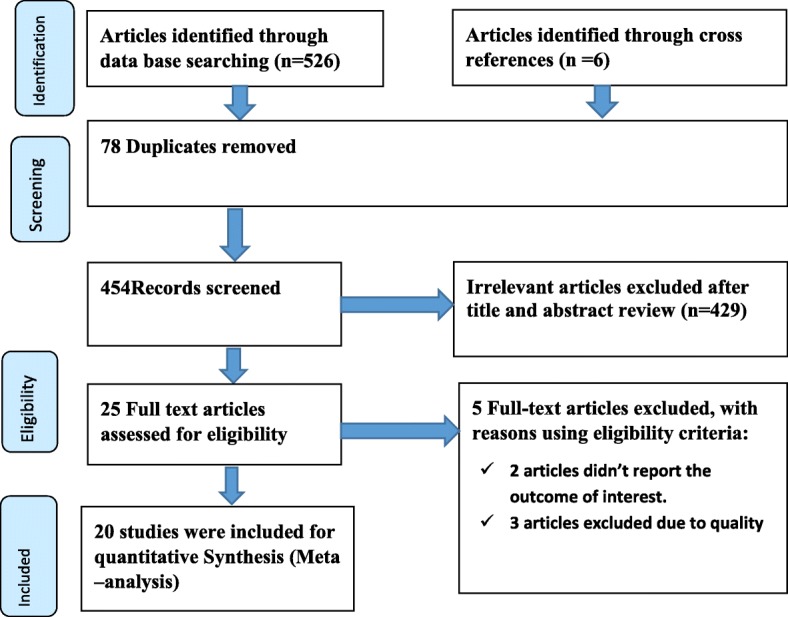
PRISMA flow diagram of included studies to estimate the pooled prevalence of anemia and its predictors among HIV infected adult individuals in Ethiopia from 2005 up to 2017

A total of 532 articles searched through the electronic (526) and supplementary (6) searches of which 78 duplicated articles were excluded. From the remaining 454 articles, 429 articles were excluded after reading of titles and abstracts based on the pre-defined inclusion criteria’s. Finally, 25 full text articles were accessed and assessed for eligibility criteria. Based on the pre-defined criteria and after critical appraisal, only 20 articles were included for the final analysis (Fig. 1).

### Characteristics of included studies

A total of twenty articles met the inclusion criteria. All the included studies were published between 2005 and 2017. Both cross sectional and cohort studies were included accordingly using an estimated sample size range from 172 [27] up to 1061 [16] adult HIV infected individual samples who were taken at Southwest parts of Ethiopia in the year 2014 and Addis Ababa city in the year 2011/2012 respectively. About 3139 men and 4920 women with a total sample of 8, 059 adult HIV infected individuals were included to estimate the pooled prevalence of anemia and its associated factors among adult HIV infected individuals (Table 1).

**Table 1:** characteristics of included studies to estimate the pooled prevalence of anemia and its predictors among adult HIV infected individuals in Ethiopia from 2005 up to 2017

ID	Region	Author	Study year	Publication year	Study design	Sampling method	Sample size	Gender distribution		Mean age In years	Measurement of anemia	Quality score
								Male	Female			
1	Addis Ababa	Tamir Z et al. [21]	2011/2012	2018	Cohort	_	394	142	252	_	Hemoglobin level	7
2	SNNP	Alamdo A et al. [3]	2006–2012	2015	Cross sectional	_	411	216	195	33.9	Hemoglobin level	6
3	Addis Ababa	Woldeamanuel G et al. [13]	2017	2018	Cross sectional	Simple random	255	148	107	40.6	Hemoglobin level	8
4	Amhara	Beyene H et al. [26]	2012/2013	2017	Cross sectional	_	528	278	250	33.69	Hemoglobin level	8
5	Amhara	Melese H et al. [18]	2015	2017	Cross sectional	Simple random	377	234	143	35.21	Hemoglobin level	8
6	Amhara	Alem M et al. [28]	2011/2012	2013	Cross sectional	Simple random	384	233	151	37	Hemoglobin level	8
7	Amhara	Deressa et al. [20]	2016	2018	Cross sectional	Systematic	320	203	117	_	Hemoglobin level	6
8	Amhara	Fiseha T et al. [23]	2010–2013	2013	Cohort	Simple random	373	233	140	34.6	Hemoglobin level	8
9	Amhara	Tesfaye Z et al. [14]	2014	2014	Cohort	_	349	218	131	34.6	Hemoglobin level	8
10	Oromia	Gedefaw L et al. [19]	2012	2013	Cross sectional	_	234	145	89	32.09	Hemoglobin level	6
11	Addis Ababa	Wolde H et al. [22]	2008–2012	2014	Cohort	Simple random	616	401	215	_	Hemoglobin level	5
12	Addis Ababa	Assefa M et al. [16]	2011/2012	2015	Cohort	Simple random	1061	632	429	_	Hemoglobin level	8
13	Amhara	Bamlaku E et al. [2]	2012/2013	2014	Cohort	_	319	202	117	_	Hemoglobin level	5
14	SNNP	Daka D et al. [15]	2005–2010	2013	Cross sectional	_	384	254	130	33.64	Hemoglobin level	7
15	Amhara	Ferede G et al. [24]	2011/2012	2013	Cohort	_	400	278	122	_	Hemoglobin level	5
16	Dire Dawa	Geleta D et al. [25]	2014	2016	Cross sectional	Simple random	425	275	150	34	Hemoglobin level	8
17	Gambella	Sahle T et al. [27]	2014	2017	Cross sectional	Systematic	172	68	104	31.95	Hemoglobin level	8
18	SNNP	Muluken W et al. [17]	2016	2018	Cross sectional	Simple random	376	195	181	_	Hemoglobin level	5
19	SNNP	Gedel G et al. (2015) [34]	2013/2014	2015	Cross sectional	Systematic	305	189	116	39.5	Hemoglobin level	8
20	Tigray	Hadgu T et al. (2013) [35]	2012	2013	Cross sectional	Systematic	376	376	_	32.5	Hemoglobin level	8

Of the total 20 articles, more than one third of studies were conducted in Amhara regional state health institutions [2, 14, 18, 20, 23, 24, 26, 28]; four studies in Addis Ababa health institutions [13, 16, 21, 22]; four studies at South Nations Nationalities and peoples of Ethiopia national regional state (SNNP) [3, 15, 17, 34]; one study at Oromia national regional state [19], one study at Gambella national regional state [27], one study at Tigray regional national state [35] and one study at Dire Dawa town administration of Ethiopia [25].

Regarding the study designs, 13 studies [3, 13, 15, 17–20, 25–28, 34, 35] and the remaining seven were cross sectional and cohort studies respectively. After critical appraisal of each articles based on JBI-MAStARI, both in peer and independently, they scored in the range of 5-up to 8 out of 9 values (see Table 1).

### Prevalence of anemia among adult HIV infected individuals in Ethiopia (Meta-analysis)

**Fig. 2:**
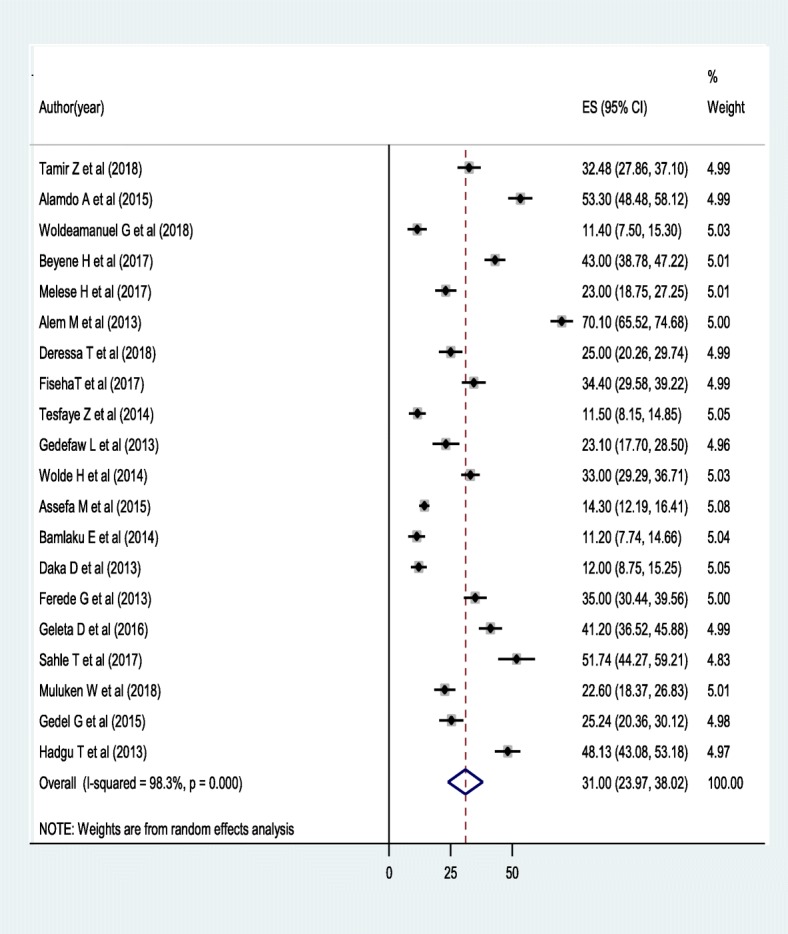
Forest plot showing the pooled prevalence of anemia among adult HIV infected individuals in Ethiopia from2005 up to 2017

As shown in the forest plot below, the pooled prevalence of anemia among adult HIV infected individuals in Ethiopia was 31.00% (95% CI: 23.94, 38.02) (Fig. 2). Substantial level of statistically significant heterogeneity was detected (I^2^ = 98%; *p* < 0.001) suggesting that the use of random effects model in estimating the pooled estimates is appropriate. The substantial magnitude of the heterogeneity also suggests the need to conduct subgroup analysis which demands identifying the sources of heterogeneity (Fig 2).

### Subgroup analysis

Since this review is exhibited with substantial heterogeneity, subgroup analysis based on study year, type of study design, study year, mean age in years, gender distribution, sample size and sampling technique they used were considered to identify the possible source of heterogeneity across studies (Table 2). However the subgroup analysis result indicated that the source of heterogeneity was not due to type of study design, study year, mean age in years, gender distribution, sample size and sampling technique they used.

**Table 2:** Sub group analysis which describes pooled prevalence of anemia and its predictors among adult HIV infected individuals in Ethiopia

Sub group		Number of included studies	Prevalence (95% CI)	Heterogeneity statistics	*P* value	I^2^
By study design	Cross sectional	13	34.54 (24.54, 44.55)	761.77	< 0.001	98.4
	Cohort	7	24.44 (16.18, 32.70)	228.57	< 0.001	97.4
Time of study year	Before 2015	16	33.65 (25.13, 42.16)	1077.02	< 0.001	98.6
	2015 and above	4	20.43 (14.12, 26.74)	26.29	< 0.001	88.6
Gender distribution	Male	12	41.21 (27.07,55.36)	774.85	< 0.001	98.6
	Female	12	34.52(24.27,44.77)	309.79	< 0.001	96.4
sampling method	Simple random	8	31.20 (18.86, 43.54)	598.36	< 0.001	98.8
	Systematic	4	37.37 (23.72, 51.02)	77.69	< 0.001	96.1
	Un-defined	8	27.64 (16.62, 38.66)	413.30	< 0.001	98.3
Mean age in years	<= 35	8	34.31 (21.75, 46.88)	432.49	< 0.001	98.4
	> 35	5	34.54 (14.59, 54.49)	422.51	< 0.001	99.1
	No report of mean age	7	24.71(17.28, 32.14)	172.93	< 0.001	96.5
Sample size	< 384	11	25.91 (18.63, 33.19)	296.43	< 0.001	98.3
	> = 384	9	37.1 (24.68,49.51)	1141.04	< 0.001	99.0

Publication bias was observed using both begg’s and egger’s test [36, 37] and the value was found to be significant at *p* value of 0.002 and 0.004 respectively. So that trim and fill meta-analysis [38] has been done to account for the publication bias. Based on this analysis, the prevalence of anemia among HIV infected individuals was 31% (95% CI: 23.97, 38.02) and no significant change was exhibited as compared with the main meta-analysis.

### Meta regression

In addition to subgroup analysis and publication bias, Meta regression was also undertaken by considering both continuous and categorical data to identify associated factors with the pooled prevalence of anemia. Sample size, study year, publication year, study design, Gender distribution, sampling technique and mean age in years for each individual studies were considered in the meta-regression. But the meta regression showed that the pooled prevalence of anemia among HIV infected adult individuals was not associated with sample size, study year, publication year, study design, Gender distribution, sampling technique and mean age in years (Table 3).

**Table 3:** Meta regression for the included studies to identify source of heterogeneity for the prevalence of anemia among HIV infected adult individuals in Ethiopia from 2005 up to 2017

Variables	Characteristics	Coefficients	P-value
Year	Publication year	−.8434435	0.672
Study year	Before 2015	Reference	Reference
	2015 and above	−13.14142	0.156
Sample	Sample size of each articles	−.0092616	0.665
Study design	Cohort	−10.0263	0.199
	Cross sectional	Reference	Reference
Gender distribution	Male	.0071367	0.883
	Female	Reference	Reference
Sampling technique	Simple random	3.11105	0.747
	Systematic	15.15263	0.385
	Undefined	Reference	Reference
Mean age in years	<=35	9.531214	0.282
	> 35	9.762604	0.328
	No report of mean age	Reference	Reference

### Associated factors of anemia among HIV infected adult individuals in Ethiopia

Low CD4 count, WHO clinical stage III&IV, OIs and low level of BMI were found to have significant association with the occurrence of anemia among HIV infected adult Individuals.

**Fig. 3:**
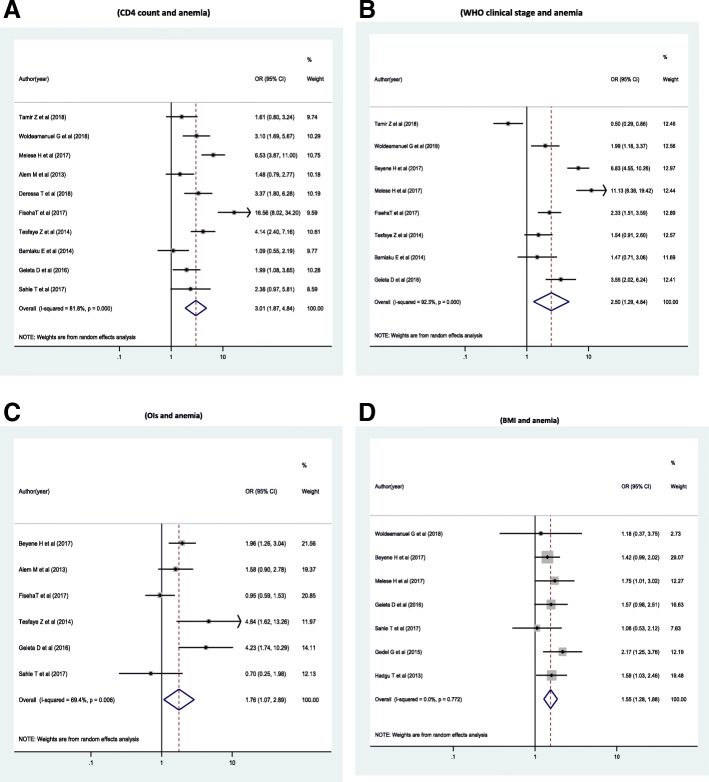
Forest plots which describe associated factors of anemia among HIV infected adult individuals in Ethiopia. **a** Forest plot which describe the association between anemia and CD4 count response among HIV infected adult individuals in Ethiopia. **b** Forest plot which describe the association between anemia and WHO clinical stage among HIV infected adult individuals in Ethiopia. **c** Forest plot which describe the association between anemia and OIs among HIV infected adult individuals in Ethiopia. **d** Forest plot which describe the association between anemia and BMI among HIV infected adult individuals in Ethiopia

The odds of developing anemia were 3 times higher among HIV infected individuals with CD4 count of < 200 cell/ul compared with HIV infected individuals with CD4 count is > = 200/ul [(OR = 3.01 (95% CI: 1.87, 4.84))] (Fig. 3a). The odds of developing anemia were 2.5 times higher among HIV infected Individuals with WHO clinical stage III &IV compared with individuals with WHO clinical stage I&II [OR = 2.5 (95% CI: 1.29, 4.84)] (Fig. 3b). The odds of developing anemia were 1.8 times higher among HIV infected Individuals with opportunistic infection had compared with individuals free of opportunistic infections [OR = 1.76 (95% CI: 1.07, 2.89)] (Fig. 3c). The odds of developing anemia were 1.6 times higher among HIV infected Individuals whose body mass index (BMI) < 18.5 kg/m^2^ had compared with those whose BMI was > = 18.5 kg/M^2^[OR = 1.55 ((95% CI: 1. 28, 1.88)](Fig. 3d).

## Discussion

This systematic review and meta-analysis was established out to estimate the pooled prevalence of anemia and its associated factors among HIV infected adult individuals in Ethiopia from 2005 [15] up 2017 [13].

According to this review, one third of HIV infected adult individuals were found to be anemic in Ethiopia. The prevalence of anemia in the current systematic review is higher than the finding of the study conducted in Tanzania [39] which estimated only severe form of anemia. The finding is also lower than the finding of the study conducted in European countries [40] and south India [41].

This variation may be due to variation in hemoglobin cut off point to define anemia across those regions. In Ethiopian studies anemia was defined if hemoglobin level was less than 11 g/dl for both men and women according to WHO definition criteria for anemia. However, in the studies conducted in European countries anemia was considered as less than or equal to 14 g/dl for men and less than or equal to 12 g/dl for women. On the other hand, anemia was considered as less than 13 g/dl for men as and less than 12 g/dl for women in the South Indian stud.

Another explanation for the difference in magnitude may be attributed to the geographic location variation where altitude difference across Ethiopia, Tanzania and European countries may change the level of hemoglobin concentration. The meta analysis results also indicated that CD4 count of HIV infected individual was found to have statistically significant association with anemia. Decrease in CD4 count of HIV infected individual leads to the increase progression to [42]. This finding is consistent with the findings reported in the study conducted in the Kathmandu [43], the united states [4], in south Africa [44], in Tanzania [39], in China [45] and Central region of Ghana [46]. This is due to patients with low Cd4 count may exposed with different opportunistic infections that in turn exposed to anemia. Hence rate of falling hemoglobin level also indicates falling of CD4 count, which is a gold standard for monitoring HIV progression [47].

The finding of current review is also consistent with the findings of studies conducted in South Africa [44], south India [41], Tanzania [39] in which WHO clinical stage III/IV of HIV, low level of body mass index (< 18.5 kg/m^2^) and different forms of opportunistic infections were an independent predictors of anemia among HIV infected individuals.

In addition, evidences also documented that individuals with advanced stage of HIV exposed to OIs [48]. These OIs also may cause dietary problems which would led to nutritional deficiencies and problems of absorbing nutrients which in turn would lead to anemia and malnutrition [49].

### Theoretical and practical implications

HIV infected adult individuals are more prone to develop concomitant infections demanding comprehensive package of health services including ART. This review evidence also indicated that those anemic HIV infected individuals deserve more attention where integrated service provision is critical to identify and manage underlying causes for anemia at early stages. Hence, specific considerations tailored to anemia screening and management needs to be taken into account during ART program design and implementation across the health system. Despite the vast investment of resources in tackling the occurrence of anemia in low and middle income countries few studies, are available to inform policy and decision making. Therefore, further studies are also recommended to generate pooled evidence at global level in general and in developing countries in particular.

### Strengths and limitations

The main strength of the current review lies in our adherence to international standardized guidelines on the conduct and reporting of systematic reviews. We included studies only from peer-reviewed English-language journals, which may have restricted our findings. Though searching was done for unpublished papers, only published studies were included.

## Conclusion

This systematic review and meta-analysis showed a high prevalence of anemia among HIV infected adult individuals in Ethiopian in which in 1/3 of HIV infected adult individuals were anemic. The review found that low CD4 count; advanced stage of the disease (WHO clinical stage III &IV), opportunistic infection and low level of BMI were found to have positive significant association with the development of anemia. Therefore, the responsible stockholders including ART clinics should strengthen the system and procedures for the early diagnosis of opportunistic infection and screening of underlying problems like under nutrition. The system should focus on technical update training on opportunistic infection diagnosis and treatment guidelines, on nutritional assessment, counseling and support (NACs), strengthening of laboratory facilities which can detect opportunistic infections early in infection. Moreover linkage of case managers, mother groups and HIV/AIDs union with ART clinic should also be strengthened for better referral linkage which can indirectly help for early screening of opportunistic infection and undernutrition. There should also be strict and frequent monitoring of HIV infected individuals CD4 count than previous in order to minimize anemia and related burdens.

## Abbreviations

BMI: Body mass index
CD4: Cluster of differentiation 4
CI: Confidence Interval
HIV: Human immuno virus
OIs: Opportunistic Infections
SNNP: South Nations Nationalities and peoples of Ethiopia
